# Supplementary material for: The differences of muscle activation in forehand serve-receiving technique of male tennis players at different skills
Source: PLoS One. 2025 May 23;20(5):e0323646. doi: 10.1371/journal.pone.0323646 (PMC12101678; doi:10.1371/journal.pone.0323646)
Supplement: Attachment — Submitted filename: Response to Reviewers.docx [file pone.0323646.s003.docx]

**Revision Notes**

We would like to express our sincere gratitude to the editors and reviewers for their time, efforts, and suggestions for our manuscript “The differences of muscle activation in forehand serve-receiving technique of male tennis players at different skills”.

**Reviewer 1**

1. Lines 15-17: The abbreviations (AG, IG) are not explicitly stated in the body of the article and are recommended to be stated when they first appear. For example, "30 male tennis players (no difference in age, height, and weight) were divided into senior (AG, n=15) and intermediate (IG, n=15) groups......"

***Response:*** Thank you for your insightful suggestion. We have revised it as suggested, please see page 2 lines 15-16.

2. Lines 24-25: The "three phases" do not specify which phases (e.g. before, during, and after the shot) and it is recommended to add a clear definition.

***Response:*** Thank you for your insightful suggestion. We have revised it as suggested, please see page 2 lines 23-24.

3. Line 32: The current literature review on muscle activation research is relatively general, citing a wide range of studies (e.g., grip, path, rotation, etc.), and the authors can focus on the blank spots in the study of muscle activation in the return ball.

***Response:*** Thank you for your insightful suggestion. We supplemented the EMG literature accordingly, please see the Introduction section.

4. Lines 62-63: Errors in the text should be corrected promptly to ensure that all references are linked accurately.

***Response:*** Thank you for your insightful suggestion. We have revised it as suggested.

5. Lines 91-92: Specific information for statistical power analyses (e.g., effect size) can be added to demonstrate the adequacy of the sample size.

***Response:*** Thank you for your insightful suggestion. We have revised it as suggested, please see page 6 line 109.

6. Lines 127-129: Although the options for wide-court zones and T-shaped/wide-court serves are mentioned, the rationale is brief. The author can provide more background information on how these settings simulate real-world match scenarios.

***Response:*** Thank you for your insightful suggestion. We have revised it as suggested, please see page 5 lines 83-97.

7.Lines 159-163: The EMG signal data processing needs to provide more details on the muscle activation solution is taken as a second-order differential equation? To provide more effective evidence, the authors may consider referring to the following relevant studies: Accurately and effectively predict the ACL force: Utilizing biomechanical landing pattern before and after-fatigue (https://doi.org/10.1016/j.cmpb.2023.107761); Adaptive neuro-fuzzy inference system model driven by the non-negative matrix factorization-extracted muscle synergy patterns to estimate lower limb joint movements (https://doi.org/10.1016/j.cmpb.2023.107848)

***Response:*** Thank you for your insightful suggestion. It is not taken as a second-order differential equation, we have revised the EMG signal data processing as suggested, please see page 10 lines 195-205.

8. Lines 218-221: "TP and PM are the main muscle groups, and these are part of the discussion. The results section should be dominated by data presentations and avoid speculative or explanatory language.

***Response:*** Thank you for your insightful suggestion. We have revised it as suggested, please see page 14 lines 253-254.

9. Line 252: Check and fix reference errors.

***Response:*** Thank you for your insightful suggestion. We have check and fix all reference.

10. Lines 310-311: The high-speed serve was studied to lead to faster muscle fatigue, which in turn affected activation patterns or athletic performance. If fatigue effects are not tested, their possible effects should be mentioned in the limitations.

***Response:*** Thank you for your insightful suggestion. We have revised it as suggested, please see page 19 lines 354-357.

11. In the conclusion section, the practical application of the research and the future research direction can be explained more clearly.

***Response:*** Thank you for your insightful suggestion. We have revised it as suggested, please see page 19 lines 362-374.

**Reviewer 2**

Dear Authors,
The Authors did a good job in writing a readable manuscript. This paper could be considered qualified to be published on “PlosOne” if the authors apply the modifications requested, "Major issues" are recommended. It is the reason why "Major revision" is my personal choice. I would be happy to review a revised version of this manuscript.
The aim of the submitted manuscript was to analyze differences in muscle activation of the right upper limb and part of the trunk and differences in stroke performance of forehand serve receiving in male tennis players of different levels of performance at different speed. This paper could be considered qualified to be published if the authors apply the modifications requested. While it is a very interesting topic, some suggestions for improving the paper are provided below:
1. The basic descriptive level adopted by author(s) does not seem compatible with interests of both researchers' biomechanical conceptual challenges and coaches' practical applications to training. The main concern refers to the weak/absent theoretical background; there is no perceivable research question or rationale for the study presented to readers.

***Response:*** Thank you for your insightful suggestion. This paper aims to analyze how to efficiently improve tennis players forehand receive performance as well as to prevent tennis injuries, and we add relevant literature and background, presenting the fundamentals as well as the issues that need to be investigated. We have revised it as suggested, please see the Introduction section.

2. Author(s) do not consider any specific difficulties or other aspects of performing these different types of tennis techniques to minimally justify the comparison;

***Response:*** Thank you for your insightful suggestion. The receive technique is the key to the start of a game and influences the tactical implementation of the game. However, current research has not focused on receive technology, the difficulty of receive technology lies in the control of the server, and the current serve machine cannot achieve the speed, ball placement, and spin of the player's serve. Therefore, in this study, we chose the player with the highest level among the participants to serve. To ensure the accuracy of serving speed and placement, before the formal experiment, we trained the server several times in advance, used high-speed cameras to test the speed of each ball to improve the accuracy of the serve, and at the same time, in the formal experiment, we regarded the data that the serve speed meets the requirements as valid data, otherwise excluded. Therefore, two serve speeds can be minimally guaranteed, and it is reasonable to compare the differences in the players' serve-receiving movements at the two serve speeds while ensuring that the serving conditions are consistent. We have revised it as suggested, please see page 7 lines 131-137.

3.On the other hand, the use of the descriptive knowledge obtained from the observed differences was not discussed in terms of applications for athletes and coaches. In short, the rationale for the study is unclear. The manuscript is limited to a biomechanical laboratory exercise to spatially and temporally describe the participants' movement.

***Response:*** Thank you for your insightful suggestion. We have added the relevant statements, please see page 19 line 362-374.

4. It is important to include more information about the balls. It is not possible to totally exclude these information from the paper (brand, company, model, etc).
***Response:*** Thank you for your insightful suggestion. We have revised it as suggested, please see page 9 lines 159-160.

5. I think much of the Procedure section needs to be rewritten to better describe procedures. I would suggest dividing into subchapters (Examples: EMG, warm-up, set-up, etc.)

***Response:*** Thank you for your insightful suggestion. We have revised the Procedure section as suggested, please see page 9 lines 162-183.

6. The following variables can’t be totally excluded from the paper: number of repetitions, serve errors, serves in, let, etc.

***Response:*** Thank you for your insightful suggestion, we did not exclude these variables, they are important. The number of repetitions in this study was determined by each participant's participation in two serve speed tests, with three valid tests collected for each speed, for a total of six repetitions, with 30-second intervals between each test, please see page 9 lines 175-178. Secondly, we treat serve errors as invalid data, and the current analyses are all for correct serves and therefore do not show specific serve errors, serves in, let.

8. Figure 1 shows the Serve-return Motion Capture setup diagram. Dimension/position of Serve placement area and dimension/position of the Serve placement area have to be justified by Authors. References about this setup need to be included to support this methodology.
***Response:*** Thank you for your insightful suggestion. We have revised it, please see page 5 lines 92-97 and Figure 1.

9. Line 124. There is a typing error (Table tennis?)

***Response:*** Thank you for your insightful suggestion. We have revised it.

10. Limitations: muscle activation of the left upper limb and part of the trunk were excluded from the present analysis
***Response:*** Thank you for your insightful suggestion. We have revised it, please see page 19 lines 357-360.

Finally, this manuscript could be considered qualified to be published if the authors apply the previous modifications, therefore “Major revision” is recommended.
