# Supplementary material for: The differences of muscle activation in forehand serve-receiving technique of male tennis players at different skills
Source: PLoS One. 2025 May 23;20(5):e0323646. doi: 10.1371/journal.pone.0323646 (PMC12101678; doi:10.1371/journal.pone.0323646)
Supplement: Attachment — Submitted filename: Response_to_Reviewers_auresp_2.docx [file pone.0323646.s004.docx]

**Revision Notes**

We would like to express our sincere gratitude to the editors and reviewers for their time, efforts, and suggestions for our manuscript “The differences of muscle activation in forehand serve-receiving technique of male tennis players at different skills”.

**Reviewer 1**

Thank you for your revised manuscript. Upon review, I noticed that the suggested references and additional review points have not been fully addressed. To ensure a more comprehensive discussion and strengthen the manuscript’s contribution, I encourage you to carefully incorporate the recommended references and revisions. Addressing these points will improve the clarity and rigor of your study.

Please let me know if any clarification is needed. Looking forward to your updated submission.

***Response:*** Thank you for your insightful suggestion. We carefully incorporate the recommended references and revisions, please see page 10 lines 196-206.

**Reviewer 3**

The study has significant flaws in experimental design, data collection, and analysis, compromising its validity and reliability. Using a manual serve instead of standardized equipment introduces subjectivity and reduces reproducibility. The reliance on a single high-speed camera limits accurate three-dimensional kinematic analysis, with no calibration or error analysis reported. EMG data processing lacks transparency, with incomplete details on noise filtering, baseline correction, and temporal characterization, resulting in a limited understanding of muscle synergy. Additionally, the absence of effect sizes and confidence intervals weakens the statistical analysis. To enhance scientific rigor, the authors should adopt standardized equipment, improve data collection methods, and ensure proper statistical reporting.

***Response:*** Thank you for your insightful suggestion. During the test, we also wanted to use standardized equipment to set up the serve conditions, the tennis serve trajectory is generated by hitting the ball quickly from high to low. However, the tennis serve machines currently on the market usually eject the ball by extruding two tracks in opposite motion, and the most important thing is that the trajectory of the serve machine is a parabola, which is very different from the manual serve trajectory. The most important thing is that the trajectory of the machine is parabolic, which is very different from the nearly straight trajectory of the manual serve. We compared the performance of a manual service with that of standardized equipment several times. In the end, considering the necessity of reproducible serve conditions, we chose a manual serve and set the conditions of ball speed, ball drop, and direction to eliminate subjectivity and increase reproducibility as much as possible.

Regarding your point that ‘the high-speed camera was not calibrated and analyzed for errors’; we agree with its importance. In this study, the high-speed camera was mainly used to differentiate between high-speed and low-speed serves, rather than accurately quantifying the absolute value of ball speeds, and was mainly used to filter valid data that matched the preset range of serve speeds (130km/h < low-serve speed < 140km/h; 160km/h < low-serve speed < 170km/h), to ensure the validity of the two types of serve speed groupings. At the same time, the setup position of the high-speed camera and the 1m marker line was completely fixed throughout the experiment, and an independent researcher was available to monitor the speed and landing point of the serve to ensure that the serving conditions were in line with the preset intervals as much as possible. Considering that the focus of this study was on the myoelectric characterization of the receiving side rather than the precise measurement of the serve speed, we did not carry out an error analysis and will adopt a more rigorous approach in future studies.

Regarding your question ‘EMG data processing lacks transparency, with incomplete details on noise filtering, baseline correction, and temporal characterization’, in this study, we used the original EMG signals filtered by a 4th order Butterworth band-pass filter (50-500Hz). Before the experiment we tested the EMG data of the forehand full power hitting action of the athletes, the maximum activation values of each muscle were selected as maximal voluntary contractions (MVC). And all the data were standardized according to the MVC Processing. All EMG signals are taken from the moment of impact as the time zero point, with a time window of -500ms to + 1000ms. The EMG amplitude increases during the stroke, and the time window from -500ms to + 500ms is taken as the stroke phase, using the ‘moment of touch ball’ as the time zero point, Before the batting phase is the preparation phase and after the batting phase is the follow-through phase, which has been added to the text, please see page 10 lines 196-206.

Regarding your point about ‘the absence of effect sizes and confidence intervals’, we have added the relevant data to the original data, please see Table 2-5.
